# Electronic cigarettes and insulin resistance in animals and humans: Results of a controlled animal study and the National Health and Nutrition Examination Survey (NHANES 2013-2016)

**DOI:** 10.1371/journal.pone.0226744

**Published:** 2019-12-31

**Authors:** Olusola A. Orimoloye, S. M. Iftekhar Uddin, Lung-Chi Chen, Albert D. Osei, Mohammadhassan Mirbolouk, Marina V. Malovichko, Israel D. Sithu, Omar Dzaye, Daniel J. Conklin, Sanjay Srivastava, Michael J. Blaha

**Affiliations:** 1 Johns Hopkins Ciccarone Center for the Prevention of Cardiovascular Disease, Johns Hopkins University School of Medicine, Baltimore, Maryland, United States of America; 2 American Heart Association Tobacco Regulation and Addiction Center, Dallas, Texas, United States of America; 3 Department of Environmental Medicine, New York University School of Medicine, Tuxedo, New York, United States of America; 4 Envirome Institute, University of Louisville, Louisville, Kentucky, United States of America; University of Pisa, ITALY

## Abstract

**Background:**

The popularity of electronic cigarettes (E-cigarettes) has risen considerably. Several studies have suggested that nicotine may affect insulin resistance, however, the impact of E-cigarette exposure on insulin resistance, an early measure of cardiometabolic risk, is not known.

**Methods and results:**

Using experimental animals and human data obtained from 3,989 participants of the United States National Health and Nutrition Examination Survey (NHANES), respectively, we assessed the association between E-cigarette and conventional cigarette exposures and insulin resistance, as modelled using the homeostatic model assessment of insulin resistance (HOMA-IR) and glucose tolerance tests (GTT). C57BL6/J mice (on standard chow diet) exposed to E-cigarette aerosol or mainstream cigarette smoke (MCS) for 12 weeks showed HOMA-IR and GTT levels comparable with filtered air-exposed controls. In the NHANES cohort, there was no significant association between defined tobacco product use categories (non-users; sole E-cigarette users; cigarette smokers and dual users) and insulin resistance. Compared with non-users of e-cigarettes/conventional cigarettes, sole E-cigarette users showed no significant difference in HOMA-IR or GTT levels following adjustment for age, sex, race, physical activity, alcohol use and BMI.

**Conclusion:**

E-cigarettes do not appear to be linked with insulin resistance. Our findings may inform future studies assessing potential cardiometabolic harms associated with E-cigarette use.

## Introduction

E-cigarettes are battery-powered devices that deliver a nicotine-containing aerosol by heating a liquid containing a solvent (vegetable glycerin, propylene glycol, or a mixture of these), flavorings, and nicotine.[1,2] These novel electronic nicotine delivery systems (ENDS) were originally proposed to be safer alternatives to conventional cigarettes, with potential utility as quit devices. However, owing to these suggestions of relative safety, aggressive marketing, and less regulation compared to conventional cigarettes,[3] there has been a steady increase in the popularity of these products in both conventional cigarette smokers and non-smokers. [4–6]

Although E-cigarette vapor may contain less total carcinogenic toxicants than conventional cigarettes,[7,8] recent studies have demonstrated that E-cigarettes affect multiple systems including immune system,[9,10] vascular functions,[11] and platelet activation[12]. Some of these adverse health effects could, at least in part, be due to inhaled nicotine.

Mechanistically, nicotine may enhance insulin resistance by causing increases in levels of insulin-antagonistic hormones including catecholamines and cortisol.[13] Furthermore, some animal studies have also suggested that nicotine directly activates an AMP-dependent protein kinase in adipose tissues, increasing the rate of lipolysis and thus promoting insulin resistance.[14]

Some human studies have suggested that cigarette smoke exposure induces insulin resistance.[15] However, the impact of nicotine-containing E-cigarette vapor on insulin resistance has not been investigated. In the absence of longitudinal human data, experimental studies and cross-sectional analyses of high-quality human datasets are critical to early understanding of possible adverse cardiometabolic effects of these new ENDS.

We therefore assessed the association between E-cigarette exposures and insulin resistance, first in a laboratory-based controlled animal study and subsequently using cross-sectional data from the National Health and Nutrition Examination Survey (NHANES).

## Methods

### Laboratory-based controlled animal study

Seven-week-old male C57BL6/J mice were obtained from the Jackson Laboratory, Bar Harbor, ME and were acclimatized for one week prior to the exposures. Mice were treated according to American Physiological Society’s *Guiding Principles in the Care and Use of Animals*. The study protocols were approved by the New York University and University of Louisville Institutional Animal Care and Use Committees. Mice were housed under pathogen-free conditions under controlled temperature and humidity, and 12h light/12h dark cycle conditions and were maintained on a standard chow diet (Rodent Diet 5010, LabDiet, St. Louis, MO; containing 4.5% fat by weight).

#### E-cigarette exposure

An automated 3-port E-cigarette aerosol generator (e~Aerosols, Central Valley, NY, USA) was used to produce E-cigarette aerosols from NJOY^®^ top fill tanks (NJOY, Inc. Scottsdale, AZ) filled with 1.6 ml e-juice in a propylene glycol/vegetable glycerin mixture (50/50 by volume, MtBakerVapor.com) without (***Vehicle***) or with nicotine (36 mg/ml; ***E-cigarette***). Starting at eight weeks of age, mice were exposed to Vehicle (n = 25) or E-cigarettes (n = 25) for 3 hours/day (between 10AM and 1PM, without food or water), 7 days/week for 12 weeks. Mice exposed to HEPA-filtered air (***Air***; n = 25) served as the control.

Each day the tanks were filled with fresh e-juice from a stock mixture and the voltage was adjusted to produce a consistent wattage (~1.96 amperes @ 4.2V) for each tank. The puff aerosols consisted of 35 ml puff volumes of 4-second duration at 30-second intervals. Each puff was mixed with filtered dilution air before entering the exposure chamber (1 m^3^). Tanks were refilled with fresh e-juice at 1.5hr into the exposure period during the pause between puffs.

#### Mainstream cigarette smoke exposure

Starting at eight weeks of age, mice were exposed for 12 weeks to mainstream cigarette smoke (MCS) generated in a multi-chamber *inExpose* system with software-controlled (FlexiWare) cigarette smoking robot (SCIREQ, Montreal, CAN). Mice were exposed daily for 6h (between 8AM and 2PM, without food or water) to MCS (n = 25) generated from burning 1 cigarette (3R4F; University of Kentucky, Center for Tobacco Reference Products, CTRP; Lexington, KY) at a time (ISO 3308:2012; i.e., 9 puffs, 35 ml/puff, 2 s/puff; 9 min/cigarette, 1 cigarette every 30 min = 12 cigarettes in 6h) or HEPA-filtered air (***Air***; n = 25). Humidified cigarettes (62 ± 1%, 24h pre-incubation with 2-way humidity packet, Boveda^®^ Inc., Wayzata, MN) were reloaded between each 2h session.

The difference in total duration of exposure to MCS (6 hours) versus E-cigarettes (3 hours) was based on the need to match nicotine exposure while limiting acute carbon monoxide exposure to the MCS-exposed mice. (i.e. the rate of combustion was limited to 2 cigarettes per hour, to avoid inducing lethargy or unconsciousness).

Immediately after the last exposure to E-cigarettes or MCS, mice were euthanized (using pentobarbital, 150 mg/kg, i.p) and their organs, blood and plasma were harvested.

#### Measurement of glycemic indices and nicotine metabolites

Measurement of glycemic indices was performed after 11–12 weeks of exposure to E-cigarettes or MCS. Blood glucose was measured in the blood withdrawn from the tail vein following 6-hour fast using Accu-chek Aviva plus blood glucose monitoring system (Roche Diagnostics, Indianapolis, IN) and plasma insulin was measured by ELISA (Mercodia AB, Uppsala, Sweden) per manufacturer’s instructions. HOMA-IR was calculated using the formula: Glucose (mg/dL) x Insulin (mU/L)/405. Glucose tolerance tests (GTT) were performed 30 min after the exposure to E-cigarettes or MCS and 6-hour fast by injecting D-glucose (1 g/kg body weight; i.p.) in sterile saline.[16–18] Nicotine, cotinine, and 3-hydroxy cotinine (3HC) in the urine were measured by liquid chromatography-mass spectrometry.[18]

**Fig 1:**
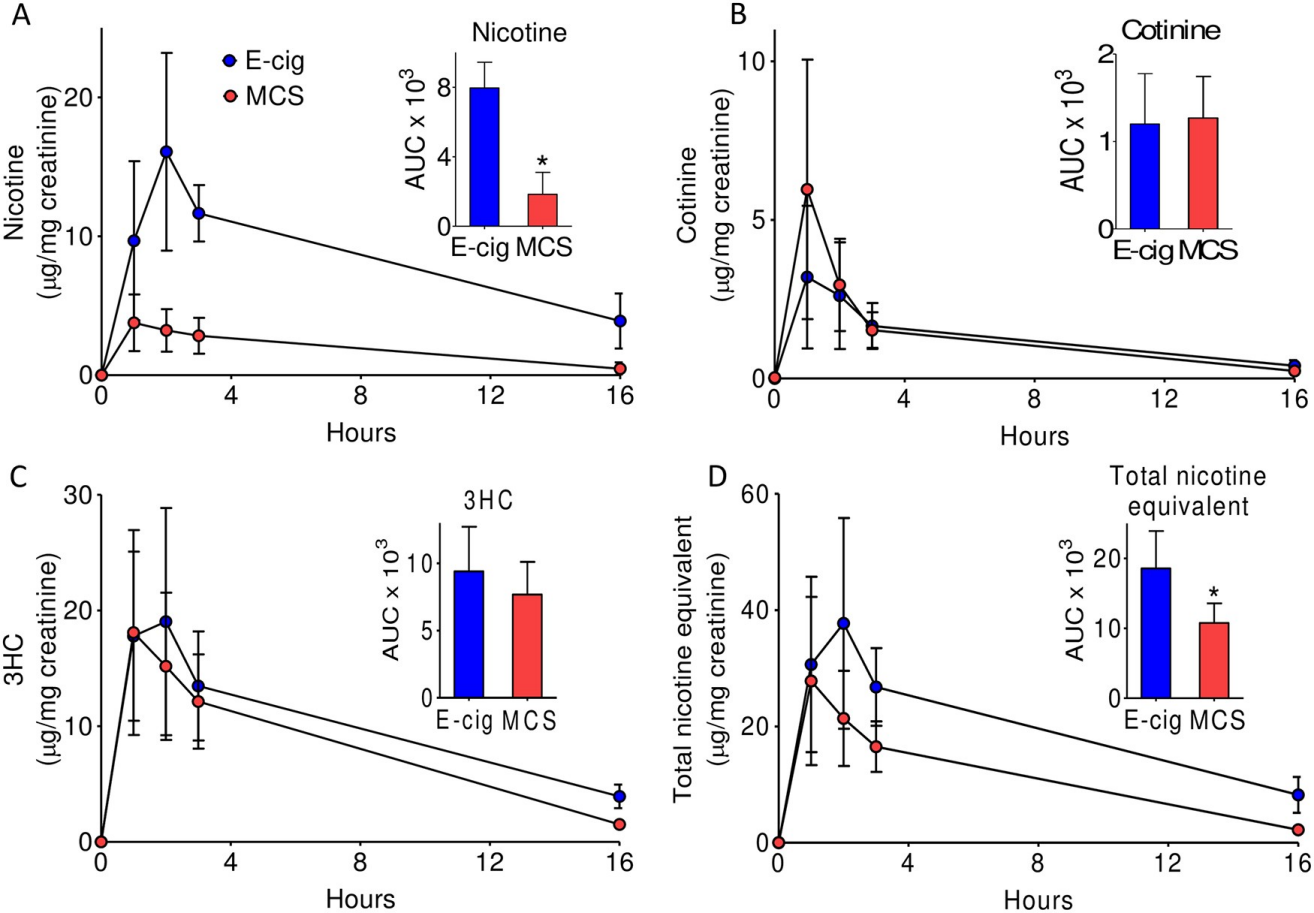
Mass spectroscopic analyses of nicotine and its metabolites in the urine of electronic cigarette- and mainstream cigarette-exposed mice. Eight week old male C57BL/6 mice were exposed to *electronic cigarette (E-cig) and mainstream cigarette smoke (MCS)* for 12 weeks. A total of 24 mice were used for the urine collection for the E-cig exposure protocol (8 mice/group) and 16 mice /group were used for MCS exposure protocol (8 mice/group). For each experimental group, two mice were housed per cage and urine was collected at the indicated time point. LC-MS/MS analyses of nicotine and its metabolites in the urine was performed as described under *Methods*. Samples were applied on UPLC and eluate were analyzed online using multiple reactions monitoring (MRM) transition. Representative elution profiles of nicotine and its metabolites cotinine, and 3HC, nicotine and cotinine are illustrated in panels **A-C** respectively. Panel **D** shows the abundance of total nicotine equivalent (nicotine+cotinine+3HC) in the samples. Insets show area under the curve for each analyte. Values are mean ± SD. *P<0.05 *versus* E-cig-exposed mice.

For measurement of urinary nicotine metabolites, mice were transferred to the metabolic cages immediately after exposure and urine was collected for 16 hours (Fig 1). Urine collection experiments were performed 2–3 days prior to euthanasia.

### Population-based human study (NHANES)

#### Study sample

We utilized data from 2013–2014 and 2015–2016 cycles of the National Health and Nutrition Examination Survey (NHANES), an annual cross-sectional, nationally representative survey of the United States civilian non-institutionalized population.[19]

Of a total of 12105 adult participants (age ≥ 18 years) across both cycles of the NHANES, 3554 had information on E-cigarette/Conventional Cigarette use status, fasting insulin, fasting glucose and glucose tolerance. Participants who had these information but responded *“Yes*” or “*borderline*” to the question *“Other than in pregnancy*, *have you been told by a doctor or health professional that you have diabetes or sugar diabetes”* (N = 134) and those who had lab confirmed or self-reported pregnancy at the time of examination (N = 5) were excluded from the analysis, leaving a total of 3415 participants in the analytical sample.

Institutional Review Board approval was not required as the NHANES represents an adequately de-identified and publicly available dataset.

#### Definition of tobacco product use categories

Current E-cigarette use was defined as use of E-cigarettes in the last 5 days. Ever cigarette-smokers were defined as lifetime smokers of at least 100 cigarettes. Ever-cigarette-smokers who answered *“everyday”* or *“some days”* to the question “*Do you now smoke cigarettes*?” were defined as current cigarette smokers. Former cigarette smokers were reclassified as current cigarette smokers if their time since quit was <1 year, while those who quit over 1 year ago were considered to be former cigarette smokers.

Based on these definitions, we defined four tobacco product use categories.

1. **Non-users** (never smokers or former cigarette smokers who do not use E-cigarettes)
2. **Sole E-cigarette users** (never smokers or former cigarette smokers with history of recent E-cigarette use)
3. **Cigarette smokers** (current cigarette smokers who do not use E-cigarettes)
4. **Dual users** (current cigarette smokers who currently use E-cigarettes)

In sensitivity analyses, we additionally handled current E-cigarette use as a dichotomous (Yes/No) variable.

#### Measurement of glycemic indices

Blood samples for fasting glucose, fasting insulin, and glucose tolerance testing (GTT) were collected by trained personnel using standardized equipment and techniques.[19] Participants were examined in the morning session and had had at least nine hours of a food fast. Participants who were taking insulin or oral medications for diabetes or those who did not drink the entire Trutol^™^ solution for GTT were excluded from testing. Plasma specimens were stored under appropriate frozen (-70°C) conditions.

Fasting glucose was reported in mg/dL, fasting insulin was reported in μU/mL, and GTT was reported in mg/dL. The Homeostatic model assessment of Insulin resistance (HOMA-IR) values were calculated thus: fasting serum insulin (μU/mL) x fasting plasma glucose (mmol/L) /22.5.[20] HOMA-IR values and GTT values were handled as log-transformed variables for analysis.

#### Statistical methods

Baseline characteristics of the study population were described across defined tobacco-product use categories. The distribution of HOMA-IR and GTT values was summarized by exposure categories and presented using box-plots.

To assess the relationship between defined tobacco product use categories and log-transformed HOMA-IR, we utilized multivariable-adjusted linear regression models, comparing all other exposure categories against a reference of non-users. Models were adjusted for age, sex, race, physical activity, body mass index, and heavy drinking. As a positive control, we also reported the multivariable-adjusted relationship between BMI (a known risk factor for cardiometabolic disease) and ln-HOMA-IR. In sensitivity analyses, we assessed the association between dichotomous E-cigarette use and ln-HOMA IR, with statistical adjustment for smoking status (never, former or current smoking). We further assessed this relationship stratified by smoking status. We repeated these analyses for GTT.

All analyses were performed using Stata 15 (StataCorp. 2017. College Station, TX) and conducted using sample weights as appropriate. For animal studies, statistical analyses were performed using SAS 9.4 software. Student’s *t* test was used to compare two sets of data. Two-way analysis of variance followed by Bonferroni post-tests was used to compare more than two experimental groups.

## Results

### Laboratory-based controlled animal study

As shown in Fig 1A, urine nicotine levels in E-cigarette-exposed mice were 4-fold higher than the urine nicotine levels in MCS-exposed mice. However, cotinine (Fig 1B) and 3HC (Fig 1C) levels in E-cigarette-exposed mice were comparable with corresponding cotinine and 3HC levels in the urine of MCS-exposed mice. The total nicotine equivalent (nicotine+cotinine+3-HC) in the urine of E-cigarette-exposed mice was 1.8-fold higher than the MCS-exposed mice (Fig 1D).

**Fig 2:**
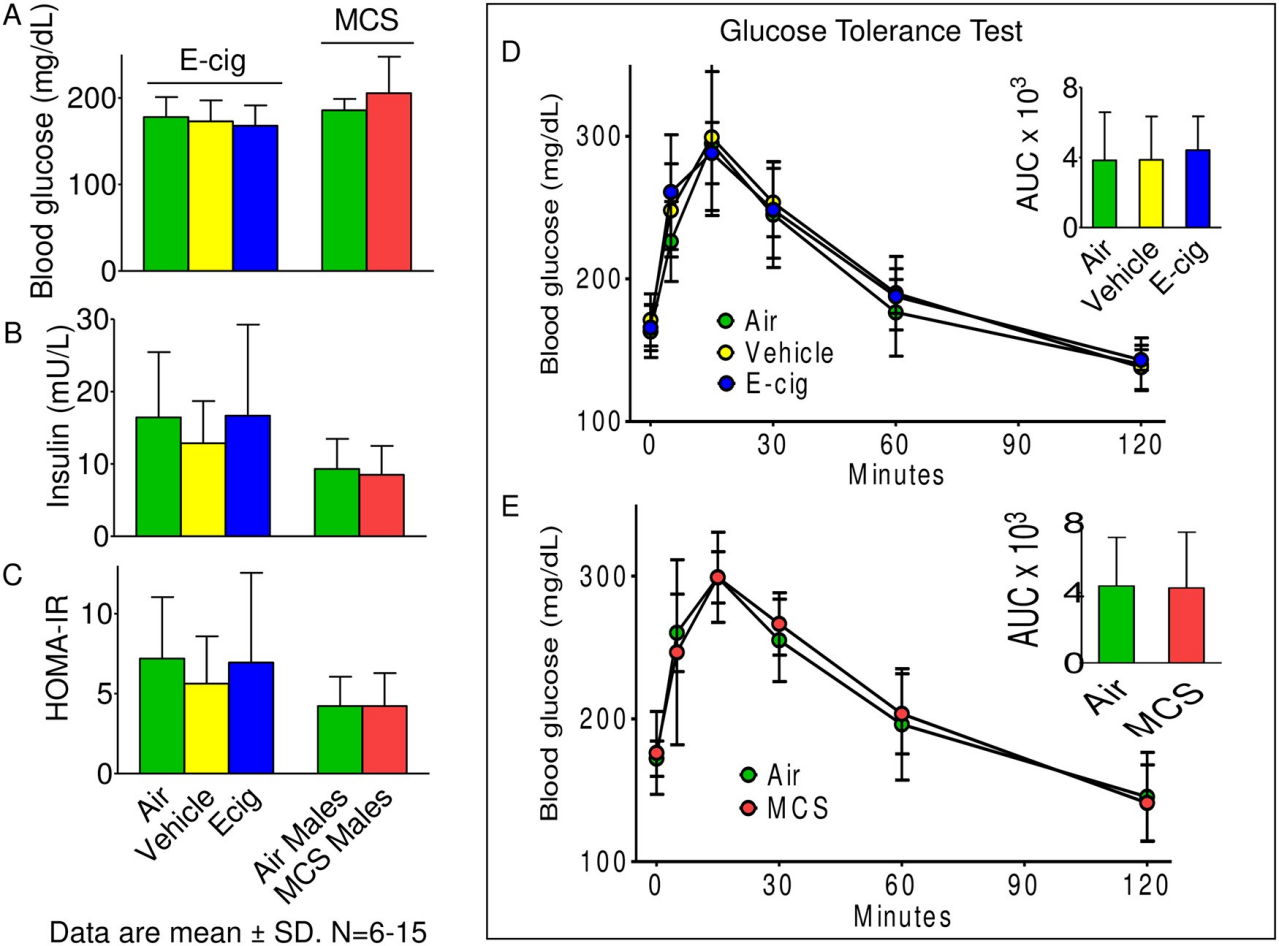
HOMA-IR levels and glucose tolerance test in E-cig- and MCS-exposed mice. Mice were exposed to E-cig or MCS for 12 weeks. For E-cig exposures propylene glycerol: vegetable glycerine 50:50, v/v was used as vehicle to aerosolize nicotine. Blood glucose (**A**) and insulin levels (**B**) were utilized to calculate HOMA-IR scores (**C**) (n = 13-15/group). Glucose (1 g/kg; i.p.) tolerance test (GTT) in mice exposed to E-cig (**D**; n = 6/group) or MCS (**E**; n = 8/group) was performed after 11–12 weeks of exposure. Insets show area under the curve for exposed mice. Values are mean ± SD.

Fasting blood glucose, insulin, and HOMA-IR levels in mice exposed to E-cigarette or MCS for 12 weeks were comparable with controls (Fig 2). Similarly, E-cigarette and MCS exposed mice had glucose tolerance comparable with air-exposed controls. Insulin-induced vasorelaxations ex vivo were comparable across all exposure groups (S1 Fig). Twelve weeks of exposure to either E-cigarette or MCS did not affect body and organ weights (S1 Table).

### Population-based human study (NHANES)

Table 1 shows the distribution of sociodemographic and risk factor characteristics according to defined exposure categories. A total of 3415 participants were studied, with 49.9% men. Non-users were more likely to be women, while sole E-cigarette users, cigarette smokers, and dual users were more likely to be men. Approximately 67% of sole E-cigarette users were less than 45 years old. Additionally, sole E-cigarette users (46.7%) were more likely to be obese compared to cigarette smokers (32.6%) and dual users (21.1%).

**Table 1:** Baseline characteristics of study population by product use status.

	Overall N = 3415	Non-users N = 2636	Sole e-cigarette users N = 30	Cigarette smokers N = 711	Dual users N = 38
**Sociodemographic characteristics (%)**					
**Sex**					
Male	49.9	47.3	60.0	58.2	68.4
Female	50.1	52.7	40.0	41.8	31.6
**Age (years)**					
18–30	23.1	23.2	36.7	21.9	31.6
30–45	25.8	24.9	30.0	29.0	29.0
45–65	31.8	29.9	30.0	39.0	26.3
> 65	19.3	22.0	3.3	10.1	13.2
**Race/Ethnicity**					
Hispanic	26.8	28.9	30.0	19.0	26.3
Non–Hispanic White	41.6	40.1	43.3	46.7	47.4
Non–Hispanic Black	17.7	15.7	10.0	25.6	13.2
Other (including multiracial)	14.0	15.4	16.7	8.7	13.2
**Educational level**					
Less than high school	21.5	19.8	13.3	27.1	39.5
High school (or equivalent)	23.2	20.7	30.0	32.4	18.4
At least some college	55.3	59.5	56.7	40.5	42.1
**Family income (per year)**					
< $20,000	22.1	18.7	24.1	34.5	20.6
$20,000 –$45,000	30.3	28.6	24.1	36.5	41.2
$45,000 - $75,000	19.6	20.9	24.1	14.6	20.6
> $75,000	27.9	31.8	27.6	14.4	17.7
**Risk factors (%)**					
**BMI categories**					
Underweight	2.0	1.8	0	2.8	5.3
Normal weight	30.5	30.0	30.0	31.5	47.4
Overweight	33.3	33.6	23.3	33.1	26.3
Obese	34.1	34.6	46.7	32.6	21.1
**Heavy drinking**	8.9	5.9	3.4	20.1	18.6
**Any Recreational Physical activity** ^†^	51.9	54.1	50.0	43.0	60.5

Heavy drinking defined as use of ≥14 alcoholic drinks/week for men, and ≥7 drinks/week for women in a typical week.

^†^ - Recreational physical activity defined as performance of moderate or vigorous physical activity for at least 10 minutes continuously in a typical week.

S2A and S2B Fig show respective box plots of the unadjusted distribution of HOMA-IR & GTT values by product use categories. Sole E-cigarette users had modestly higher median HOMA-IR values compared to all other categories, including cigarette smokers and dual users. However, the differences across groups were not statistically significant (p = 0.17). Similarly, there were no significant differences in 2-hour glucose tolerance test measures across product use categories (p = 0.51).

In all tested models, we found no significant association between sole E-cigarette use and HOMA-IR or GTT. However, cigarette smoking was negatively associated with GTT, but not HOMA-IR in fully adjusted models. (Table 2) Beta coefficients of the multivariable adjusted association of BMI and glycemic indices were positive, and statistically significant (β_HOMA-IR_ = 0.07, 95% CI [0.068–0.072], p value <0.001; β_GTT_ = 0.01, 95% CI [0.008–0.012], p-value <0.001).

**Table 2:** Multivariable-adjusted association between product use categories and log-transformed HOMA-IR & GTT.

Tobacco product category	β-coefficient (95% Confidence interval)
HOMA-IR	
**Non-users**	**REF**
Sole E-cigarette users	0.20 (-0.09–0.49)
Cigarette smokers	-0.01 (-0.08–0.05)
Dual users	-0.13 (-0.43–0.16)
GTT	
**Non-users**	**REF**
Sole E-cigarette users	-0.05 (-0.21–0.11)
Cigarette smokers	**-0.08 (-0.12–-0.05)**
Dual users	**-0.12 (-0.24–-0.004)**

Model adjusted for age, sex, race, physical activity, heavy drinking and BMI.

As a positive control, we assessed the multivariable-adjusted association of BMI with HOMA-IR & GTT in each of these models. Beta coefficients were positive, (β_HOMA-IR_ = 0.07, 95% CI [0.064–0.072], p value <0.001; β_GTT_ = 0.01, 95% CI [0.008–0.012], p-value <0.001)

In supplementary analyses, we found no relationship between dichotomous E-cigarette use and HOMA-IR or GTT in multivariable-adjusted models additionally adjusted for smoking status (S2 Table). Similarly, in multivariable-adjusted models stratified by smoking status, E-cigarette use was not associated with HOMA-IR or GTT in never smokers, former smokers, or current smokers. (S3 Table).

## Discussion

In both a 12-week laboratory-based animal study and a cross-sectional nationally representative population-based human study, we found that E-cigarette exposure (or use) was not associated with insulin resistance, as measured using HOMA-IR and GTT. To our knowledge, this is the first study to explore the possible associations between E-cigarette exposure and insulin resistance in human subjects or experimental animals.

According to the 2018 National Academies of Sciences, Engineering and Medicine (NASEM) consensus report on the “*Public Health Consequences of E-cigarettes*”, little is known about the cardiometabolic effects of E-cigarettes.[1] Our study therefore fills an important gap by assessing the relationship between E-cigarette exposure and an important measure of cardiometabolic risk, closely linked to hard cardiovascular disease outcomes.

While our study showed no effect on HOMA-IR and GTT, further studies on other cardiometabolic risk domains are needed, particularly longitudinal human studies. Nonetheless, we believe that animal studies and cross-sectional studies as ours are important, as they may be the first signals of potential harm due to use of these products. We believe that our study is therefore important in guiding further studies of E-cigarettes, funding priorities, and regulatory actions.

A major strength of our study is that we studied the relationship between E-cigarette exposures and insulin resistance in both well-characterized animal models and in high-quality nationally representative human studies. In our animal studies, urinary cotinine and 3HC levels in E-cigarette-exposed mice were comparable with MCS-exposed mice; whereas urinary nicotine levels in E-cigarette-exposed mice were significantly higher than in MCS-exposed animals. Nonetheless, HOMA-IR and GTT data in E-cigarette-exposed mice were comparable with MCS-exposed mice, suggesting that even higher delivery of nicotine by E-cigarette did not induce insulin resistance under our experimental conditions.

Limitations include the cross-sectional nature of our human study, which limits our ability to draw causal inferences, and the lack of detailed characterization of the frequency, chronicity and intensity of exposure to E-cigarette use, which may lead to some residual confounding. For animal studies, we only used young male mice and HOMA-IR and GTT were measured only at a single (12-week) time point. However, since female mice are generally more resistant to inhalation exposures and cardiotoxicity, noxious effects, if present are more likely to be readily demonstrable in male mice than female mice, thus making the former an appropriate group for initial testing of the effects of e-cigarette exposure. [21]

In conclusion, E-cigarettes do not appear to be associated with insulin resistance, an early measure of cardiometabolic risk. These results must be coupled with ongoing and future studies investigating potential cardiovascular toxicity in other domains.

## Supporting information

S1 Fig Exposure to E-cigarette aerosol without and with nicotine had no effect on insulin-induced vasorelaxation in isolated mouse aorta. (DOCX)

S2 Fig Box plots of the distribution of HOMA-IR and GTT, respectively, by product use category. (DOCX)

S1 Table Body and organ weights of mice chronically exposed to E-cigs or mainstream cigarette smoke. (DOCX)

S2 Table Association between electronic cigarette use and log-transformed markers of insulin resistance, adjusted for smoking status, NHANES 2013–2016. (DOCX)

S3 Table Association between electronic cigarette use and log-transformed markers of insulin resistance stratified by smoking status, NHANES 2013–2016. (DOCX)
