# Supplementary material for: Electronic cigarettes and insulin resistance in animals and humans: Results of a controlled animal study and the National Health and Nutrition Examination Survey (NHANES 2013-2016)
Source: PLoS One. 2019 Dec 31;14(12):e0226744. doi: 10.1371/journal.pone.0226744 (PMC6938328; doi:10.1371/journal.pone.0226744)
Supplement: S1 Fig — (DOCX) [file pone.0226744.s001.docx]

**S1 Fig**: *Exposure to E-cigarette aerosol without and with nicotine had no effect on insulin-induced vasorelaxation in isolated mouse aorta*.  Aortic rings were equilibrated to ≈9.8 mN resting tension and tested for viability over 1h. When stable, rings were pre-contracted with phenylephrine (PE; 10 µM) and relaxed with cumulative concentrations of insulin (0.01 mU/mL-10 mU/mL -R). Values = means ± SE (n=15/group).
