# Supplementary figures and images for: Electronic cigarettes and insulin resistance in animals and humans: Results of a controlled animal study and the National Health and Nutrition Examination Survey (NHANES 2013-2016)
Source: PLoS One. 2019 Dec 31;14(12):e0226744. doi: 10.1371/journal.pone.0226744 (PMC6938328; doi:10.1371/journal.pone.0226744)

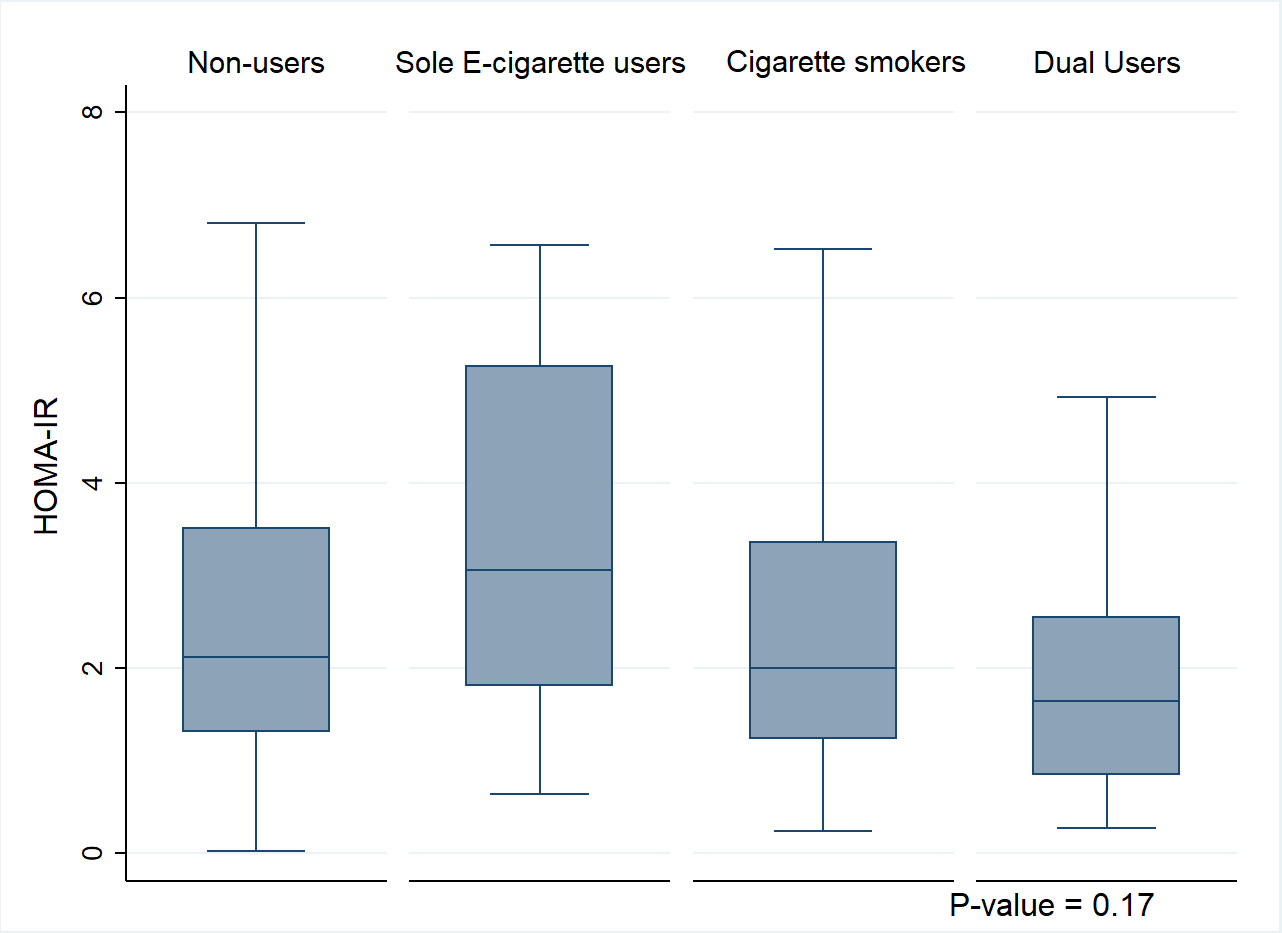


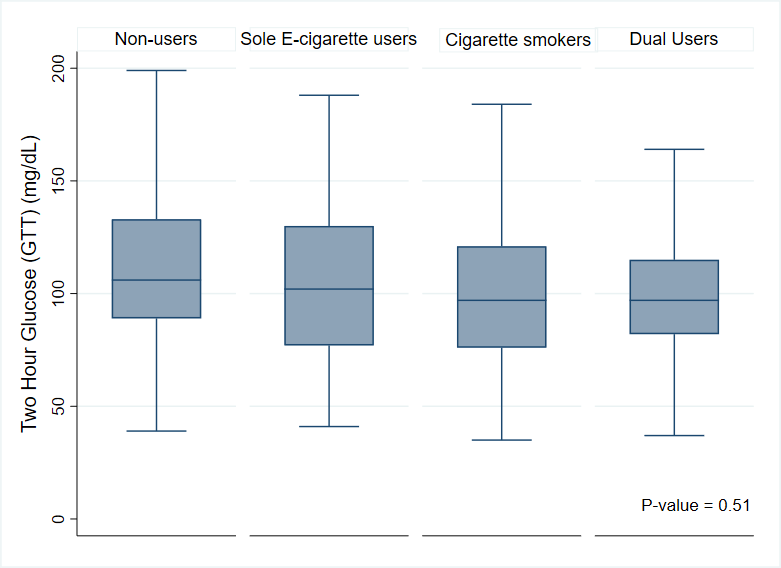


**Fig S2:** **Box plots of the distribution of HOMA-IR and GTT by product use category**

Supplement: S2 Fig — (DOCX) [file pone.0226744.s002.docx]
