# Supplementary material for: Electronic cigarettes and insulin resistance in animals and humans: Results of a controlled animal study and the National Health and Nutrition Examination Survey (NHANES 2013-2016)
Source: PLoS One. 2019 Dec 31;14(12):e0226744. doi: 10.1371/journal.pone.0226744 (PMC6938328; doi:10.1371/journal.pone.0226744)
Supplement: S1 Table — (DOCX) [file pone.0226744.s003.docx]

**S1 table.** **Body and organ weights of mice chronically exposed to E-cig or MCS**

| **Parameters** | **E-cig exposure** | | | **MCS exposure** | |
| --- | --- | --- | --- | --- | --- |
|  | Air | Vehicle | E-cig | Air | MCS |
| Initial body weight (g) | 24.3±1.3 | 25.7±2.4 | 26.2±1.7 | 23.9±1.4 | 22.8±1.3 |
| Terminal body weight (g) | 26.3±1.5 | 26.2±2.2 | 27.1±2 | 27.9±1.8 | 26.1±1.2 |
| Heart weight (g) | 0.137±0.007 | 0.134±0.012 | 0.14±0.012 | 0.149±0.017 | 0.14±0.009 |
| Heart (% body weight) | 0.52±0.02 | 0.51±0.02 | 0.52±0.03 | 0.532±0.044 | 0.536±0.04 |
| Lungs (g) | 0.164±0.01 | 0.165±0.011 | 0.164±0.014 | 0.25±0.08 | 0.26±0.09 |
| Lungs (% body weight) | 0.62±0.03 | 0.63±0.04 | 0.61±0.04 | 0.89±0.32 | 1.01±0.36 |
| Liver weight (g) | 1.3±0.11 | 1.42±0.23 | 1.32±0.17 | 1.68±0.28 | 1.51±0.28 |
| Liver (% body weight) | 4.86±0.35 | 5.47±1.19 | 4.26±1.74 | 6±0.8 | 5.8±1.1 |
| Kidneys (g) | 0.35±0.02 | 0.35±0.03 | 0.37±0.04 | 0.401±0.043 | 0.38±0.037 |
| Kidneys (% body weight) | 1.33±0.06 | 1.34±0.05 | 1.38±0.07 | 1.43±0.09 | 1.46±0.13 |
| Spleen (g) | 0.081±0.006 | 0.083±0.014 | 0.09±0.013 | 0.09±0.008 | 0.085±0.009 |
| Spleen (% body weight) | 0.31±0.02 | 0.32±0.03 | 0.33±0.04 | 0.32±0.02 | 0.33±0.04 |

Values are mean ± SD. N=8-15 per group
