# Supplementary material for: Electronic cigarettes and insulin resistance in animals and humans: Results of a controlled animal study and the National Health and Nutrition Examination Survey (NHANES 2013-2016)
Source: PLoS One. 2019 Dec 31;14(12):e0226744. doi: 10.1371/journal.pone.0226744 (PMC6938328; doi:10.1371/journal.pone.0226744)
Supplement: S2 Table — (DOCX) [file pone.0226744.s004.docx]

**S2 Table.** Association between electronic cigarette use and log-transformed markers of insulin resistance, adjusted for smoking status, NHANES 2013-2016. *

|  | **Model 1 ^a^**  **β-coefficient (95% confidence interval)** | **Model 2 ^b^**  **β-coefficient (95% confidence interval)** |
| --- | --- | --- |
|  | HOMA-IR | |
| E-cigarette non-users | REF | REF |
| E-cigarette users | -0.03 (-0.29 – 0.23) | 0.04 (-0.17 – 0.26) |
|  | GTT | |
| E-cigarette non-users | REF | REF |
| E-cigarette users | -0.04 (-0.14 – 0.05) | -0.05 (-0.16 – 0.07) |

*- E-cigarette use defined as a dichotomous (Yes/No) variable

a – Model adjusted for age, sex, race and cigarette smoking status (never, former or current smoking)

b – Model adjusted for age, sex, race, physical activity, BMI, cigarette smoking status (never, former or current smoking), and heavy drinking.
