# Supplementary material for: Electronic cigarettes and insulin resistance in animals and humans: Results of a controlled animal study and the National Health and Nutrition Examination Survey (NHANES 2013-2016)
Source: PLoS One. 2019 Dec 31;14(12):e0226744. doi: 10.1371/journal.pone.0226744 (PMC6938328; doi:10.1371/journal.pone.0226744)
Supplement: S3 Table — (DOCX) [file pone.0226744.s005.docx]

|  | **Model 1**  **β-coefficient (95% Confidence interval)** | | | **Model 2**  **β-coefficient (95% Confidence interval)** | | |
| --- | --- | --- | --- | --- | --- | --- |
|  | Never smokers | Former smokers | Current smokers | Never smokers | Former smokers | Current smokers |
|  | **HOMA-IR** | | | | | |
| E-cigarette non-users | REF | REF | REF | REF | REF | REF |
| E-cigarette users | **0.53 (0.13 – 0.93)** | 0.10 (-0.27 – 0.47) | -0.37 (-0.78 – 0.02) | 0.22 (-0.36 – 0.81) | 0.26 (-0.03 – 0.55) | -0.25 (-0.52 – 0.19) |
|  | **GTT** | | | | | |
| E-cigarette non-users | REF | REF | REF | REF | REF | REF |
| E-cigarette users | -0.01 (-0.20 - 0.17) | -0.001 (-0.18 – 0.18) | -0.08 (-0.23 – 0.07) | -0.06 (-0.18 – 0.07) | -0.003(-0.20 - 0.19) | -0.10 (-0.26 – 0.05) |

**S3 Table**: Association between electronic cigarette use and log-transformed markers of insulin resistance stratified by smoking status, NHANES 2013-2016. *

*- E-cigarette use defined as a dichotomous (Yes/No) variable

Model 1 adjusted for age, sex, and race.

Model 2 adjusted for age, sex, race, physical activity, BMI, and heavy drinking.
